# The Mediating Role of Catastrophizing in the Relationship Between Emotional Clarity and Posttraumatic Stress Symptoms Among Earthquake Survivors in Korea: A Cross-Sectional Study

**DOI:** 10.3389/fpsyg.2020.01114

**Published:** 2020-06-17

**Authors:** Ha Rin Kwon, Yookyung Eoh, Soo Hyun Park

**Affiliations:** ^1^Department of Psychology, Yonsei University, Seoul, South Korea; ^2^Yongmoon Graduate School of Counseling Psychology, Seoul, South Korea

**Keywords:** earthquake, posttraumatic stress, emotional clarity, catastrophizing, emotion regulation

## Abstract

Difficulties in emotion regulation reportedly contribute to the development and maintenance of PTSD following exposure to natural disasters. Based on the extended process model of emotion regulation, the present study hypothesized that maladaptive emotion regulation strategies will mediate the relationship between emotional clarity and posttraumatic stress symptoms in a sample of earthquake survivors. A total of 195 adult residents of Gyeongju and Pohang, southeastern coastal cities in Korea, who had experienced recent earthquakes participated in an online survey study. They completed questionnaires assessing emotional clarity, emotion regulation, and posttraumatic stress symptoms a year and 10 months after the Gyeongju earthquake and 7 months after the Pohang earthquake. Bootstrapping procedures were used to test for a mediation effect. The results suggest that emotional clarity was indirectly associated with posttraumatic stress symptoms through maladaptive emotion regulation strategies, especially catastrophizing. The findings suggest that individuals with low emotional clarity tend to use maladaptive strategies, catastrophizing in particular, which may contribute to posttraumatic stress symptoms. This may reflect the mechanism underlying emotional clarity and offer suggestions for target of treatment in the management of long-term psychological difficulties in earthquake survivors. Replication of the current results in a sample of patients diagnosed with PTSD is necessary to better understand the development and progression of the disorder, as well as effective interventions for PTSD.

## Introduction

Unprecedented earthquakes occurred between September 2016 and November 2017 in the Republic of Korea near Gyeongju and Pohang, two southeastern coastal cities in the province of Gyeongbuk that are separated by a distance of only 25 miles. The Gyeongju earthquake was recorded to be the strongest earthquake in Korean history, measuring 5.8 on the magnitude scale, and the Pohang earthquake was the second strongest earthquake, measuring 5.4. Following the earthquakes, 158 residents were physically injured, and 1,908 survivors suffered losses (Ministry of the Interior and Safety, 2018). Because Korea was generally considered to be removed from the threat of severe earthquakes, the psychological shock experienced by residents was that much greater. Following the earthquakes, 2,498 and 9,086 cases were referred for psychotherapy in Gyeongju and Pohang, respectively, with 425 reported high-risk cases (Ministry of the Interior and Safety, 2018), suggesting the significant psychological impact of the disaster on the residents.

Natural disasters such as earthquakes are unpredictable, uncontrollable, and traumatic events that affect individuals in diverse and extensive ways. Individuals who experience natural disasters report psychological distress such as depression, anxiety, and stress, and such responses in the acute stage following a disaster are typically considered natural and normal (Myers and Wee, 2005). While most disaster survivors recover from the impact of trauma within a period ranging from several months to 1 or 2 years (Bonanno et al., 2010), some survivors may report more persistent mental health problems. Among such problems, posttraumatic stress disorder (PTSD) is the most prevalent disorder following natural disasters (Neria et al., 2008). Because such psychological problems result in considerable social and individual cost (Trautmann et al., 2016), much effort has been dedicated to the identification of potential predictive factors of PTSD following natural disasters (Ozer et al., 2003).

One of the most widely examined factors is emotion regulation (Eftekhari et al., 2009; Bardeen et al., 2013). According to the extended process model of emotion regulation (Gross, 2015), emotion regulation is a complex process consisting of three stages – identification, selection, and implementation. The identification stage is an important first step that will affect subsequent processes, and it requires “emotional clarity,” which refers to the ability to identify, understand, and discriminate one’s emotions (Salovey et al., 1995). Based on the identified emotions, individuals select appropriate emotion regulation strategies and implement the selected strategies. When they succeed in this process, individuals can maintain emotional balance even in emotionally intense situations, but when they fail, they may experience psychological distress.

In this context, unexpected disasters like earthquakes are specific situations wherein individuals confront substantial demand for emotion regulation (Mennin, 2005). Disasters may trigger diverse and extreme degrees of emotions such as fear, helplessness, and horror, and individuals who experience such emotions need to identify them correctly and choose emotion regulation strategies appropriately. If emotion regulation fails, the intense negative emotions can ultimately lead to long-term psychological problems such as PTSD (Tull et al., 2007; Amstadter and Vernon, 2008).

Many studies have empirically demonstrated that difficulties in each stage of the emotion regulation process following traumatic events may contribute to the development of PTSD. For example, when emotional clarity is low, the negative impact of traumatic events was significantly greater (Shipman et al., 2005; Viana et al., 2018). Another study showed that implementing maladaptive emotion regulation strategies (e.g., emotional avoidance) as a response to emotional difficulties can lead to PTSD (Tull et al., 2011).

Although such findings contribute to understanding the role of emotion regulation following traumatic events, they also hold several limitations. First, previous studies have primarily focused on the effect of emotional clarity on interpersonal trauma (e.g., child neglect, sexual assault), and only a few studies have targeted natural disasters. However, given that the association between low emotional clarity and psychological problems such as depression was stronger when more intense negative affect is experienced (Kennedy et al., 2010; Stange et al., 2013; Vine and Marroquín, 2018), emotional clarity may play just as important a role in natural disasters. Moreover, although emotion regulation is an integrated and systematic process consisting of clear emotion identification and subsequent selection and implementation of emotion regulation strategies, most studies did not address emotional clarity and emotion regulation strategy simultaneously. Only one study suggested the association between emotional clarity and emotion regulation strategies by examining the interaction effect of emotional clarity and cognitive reappraisal in predicting PTSD (Boden et al., 2012). A few recent studies have begun to investigate the sequential effect of emotion regulation in depression using a mediation model (Vine and Aldao, 2014; Boden and Thompson, 2015), but no studies have found the mediation effect of emotion regulation strategies in the relationship between emotional clarity and PTSD. In particular, no studies have identified which *specific* maladaptive emotion regulation strategies play a more significant mediating role.

The current study thus examined the mediating role of maladaptive emotion regulation strategies on the relationship between emotion clarity and posttraumatic stress symptoms in survivors of the Gyeongbuk earthquakes. Moreover, in order to examine the unique relationship between the variables, risk factors operating before or during the earthquake, such as prior psychological problems, prior trauma exposure, physical injury, and perceived life threat (Brewin et al., 2000), were statistically controlled. The study hypothesized that maladaptive emotion regulation strategies will fully mediate the relationship between emotional clarity and posttraumatic stress symptoms, even after controlling the other risk factors.

## Method

### Participants

The sample consisted of adult residents of Gyeongju and Pohang who had experienced the recent earthquakes. From a total of 216 returned datasets, 21 individuals did not complete the questionnaires thoroughly, and thus data of 195 participants were analyzed. The final sample consisted of 87 men and 108 women with a mean age of 38.69 (SD = 10.27). The majority of participants resided in Pohang (*n* = 146). A total of 14 participants reported pre-disaster psychological difficulties, and 38 participants reported past trauma other than the recent earthquake. Exposure to personal physical injury during the earthquake was reported by 17 participants, while 47 reported exposure to others’ physical injury. A total of 114 reported experience of threat to life, and 113 reported exposure to threat of death in others during the earthquakes. Table 1 presents the participants’ demographic and disaster-related characteristics.

**TABLE 1:** Demographic and disaster-related characteristics of participants (*N* = 195).

**Characteristics**	**Male (%)**	**Female (%)**	***n* (%)**
**Age (years)**			
19–29	18 (20.7)	23 (21.3)	41 (21.0)
30–39	26 (29.9)	48 (44.4)	74 (37.9)
40–49	22 (25.3)	24 (22.2)	46 (23.6)
50–69	21 (24.1)	13 (12.0)	34 (17.4)
**Educational attainment**			
High school graduate or less	12 (13.8)	27 (25.0)	39 (20.0)
College student	3 (3.4)	11 (10.2)	14 (7.2)
Undergraduate degree	64 (73.6)	62 (57.4)	126 (64.6)
Graduate degree	8 (9.2)	8 (7.4)	16 (8.2)
**Self-reported socioeconomic status**			
Low	27 (31.0)	38 (35.2)	65 (33.4)
Average	52 (59.8)	61 (56.5)	113 (57.9)
High	8 (9.2)	9 (8.3)	17 (8.7)
**Marital status**			
Single	37 (42.5)	41 (38.0)	78 (40.0)
Married	50 (57.5)	67 (62.0)	117 (60.0)
**Employment**			
Employed	71 (81.6)	71 (65.6)	142 (72.8)
Unemployed	16 (18.4)	37 (34.4)	53 (27.2)
**Area of residence**			
Gyeongju	25 (28.7)	24 (22.2)	49 (25.1)
Pohang	62 (71.3)	84 (77.8)	146 (74.9)
**Prior psychological problems**			
Yes	5 (5.7)	9 (8.3)	14 (7.2)
No	82 (94.3)	99 (91.7)	181 (92.8)
**Prior trauma exposure**			
Yes	22 (25.3)	16 (14.8)	38 (19.5)
No	65 (74.7)	92 (85.2)	157 (80.5)
**Physical injury of self**			
Yes	10 (11.5)	7 (6.5)	17 (8.7)
No	77 (88.5)	101 (93.5)	178 (91.3)
**Physical injury of others**			
Yes	26 (29.9)	21 (19.4)	47 (24.1)
No	61 (70.1)	87 (80.6)	148 (75.9)
**Perceived life threat – self**			
Yes	37 (42.5)	77 (71.3)	114 (58.5)
No	50 (57.5)	31 (28.7)	81 (41.5)
**Perceived life threat – others**			
Yes	55 (63.2)	58 (53.7)	113 (57.9)
No	32 (36.8)	50 (46.3)	82 (42.1)

### Procedures

A study participation notice was posted on the recruitment announcement board of an online research participation platform (“dataSpring”) in Gyeongju and Pohang. Questionnaires were administrated online between June 5 and 15, 2018, which is a year and 10 months after the Gyeongju earthquake and 7 months after the Pohang earthquake, to individuals who voluntarily consented to participate. The participants were provided with 1,000 points (approximately 1USD) as a reward. The present study was approved by the university’s institutional review board.

### Measures

#### Trait Meta-Mood Scale

To measure emotional clarity, Trait Meta-Mood Scale (TMMS) (Salovey et al., 1995) was administered. The scale consists of 21 items measured on a five-point Likert scale, with five items measuring attention to emotion, 11 items measuring emotional clarity, and five items measuring expectation regarding improvement in emotion. From the Korean version of the TMMS (Lee and Lee, 1997), the 11 items assessing emotional clarity were used in the present study. In the validation study by Lee and Lee (1997), Cronbach’s α of the emotional clarity subscale was 0.86, and that of the present study was 0.76.

#### Posttraumatic Diagnostic Scale

To measure posttraumatic stress symptoms, the Korean version of the Posttraumatic Diagnostic Scale (PDS-K; Nam et al., 2010) was administered. PDS-K was developed by translating and validating the Posttraumatic Diagnostic Scale (PDS; Foa et al., 1997) with the goal of measuring the severity of PTSD symptoms and diagnosing PTSD. PDS-K consists of four chapters: chapters 1 and 2 assess traumatic events and severity of distress, chapter 3 measures the frequency of symptoms during the 1-month period following the traumatic event, and chapter 4 measures how the symptoms are contributing to the individual’s maladjustment. The present study assessed posttraumatic stress symptoms using the 17 items of chapter 3. Cronbach’s α of the scale was 0.95 in the Korean validation study (Nam et al., 2010) and 0.94 in the current study.

#### Cognitive Emotion Regulation Questionnaire

Cognitive Emotion Regulation Questionnaire (CERQ), developed by Garnefski and Kraaij (2007), measures cognitive emotion regulation strategies. Ahn et al. (2013) validated the Korean version of CERQ. The 35-item K-CERQ consists of two subscales, namely adaptive (*acceptance, refocus on planning, putting into perspective, positive refocusing, and positive reappraisal*) and maladaptive (*self-blame, other-blame, rumination, catastrophizing*) emotion regulation strategies. In the present study, only the maladaptive emotion regulation strategy subscale was analyzed. Ahn et al. (2013) reported Cronbach’s α of 0.83 for self-blame, 0.87 for other-blame, 0.76 for rumination, and 0.79 for catastrophizing subscales. Cronbach’s α values in the present study were 0.80 for self-blame, 0.87 for other-blame, 0.76 for rumination, 0.80 for catastrophizing, and 0.91 for the whole subscale of maladaptive emotion regulation strategies.

#### Factors Operating Before or During the Earthquake

Participants were asked whether they had experienced any psychological problems before the earthquake and were asked to choose “yes” or “no.” They were also asked whether they had experienced any other trauma before the earthquake in a “yes” or “no” question. Finally, using Chapter 2 of the PDS-K, “yes” or “no” questions regarding the experience of physical injury and threat to life at the time of the earthquake were completed.

### Data Analysis

There were no missing data. For data analyses, SPSS 24.0 and PROCESS macro were used. First, Cronbach’s α values were calculated to examine the internal consistency of the measures. Second, descriptive statistics and correlation analysis between the variables were conducted. Correlational coefficients were computed using Pearson correlation analyses. Third, to rule out the potential effects of gender and age, *t*-tests and ANOVA, as well as Bonferroni *post hoc* analysis, were conducted to examine gender and age differences prior to the mediation analysis. When significant differences across gender and age groups were found, gender and age were statistically controlled in subsequent mediation analyses.

Lastly, PROCESS macro for SPSS (Hayes, 2013) was conducted using bootstrapping to examine the effect of emotional clarity on posttraumatic stress symptoms and the mediating effect of each of the four maladaptive emotion regulation strategies on the relationship between the variables. It was considered as significant if 95% confidence intervals (CI) did not include 0. Factors operating before or during the trauma (e.g., prior psychological problems, physical injury) were controlled based on the results of previous studies that reported these factors as risk factors for PTSD.

## Results

### Descriptive Statistics and Correlations Between Measures

Table 2 shows the mean and standard deviation of each variable as well as the correlation coefficients between the variables. In the present study, 13.8% (*n* = 27) of the participants reported a score of 20 or higher on the PDS-K, indicative of significant posttraumatic stress symptoms (Nam et al., 2010). Results of correlational analysis found a significant correlation between all of the variables except for the correlation between emotional clarity and posttraumatic stress symptoms.

**TABLE 2:** Descriptive statistics and correlation coefficients of measured variables.

**Variable**	**1**	**2**	**3**	**4**	**5**	**6**
Emotional clarity	–	–	–	–	–	–
Self-blame	−0.26***	–	–	–	–	–
Other-blame	−0.21**	0.43***	–	–	–	–
Rumination	−0.24**	0.63***	0.50***	–	–	–
Catastrophizing	−0.28***	0.49***	0.48***	0.73***	–	–
Posttraumatic stress	–0.09	0.29***	0.34***	0.50***	0.60***	–
*M*	38.69	7.49	8.55	9.10	8.21	8.44
SD	5.24	3.40	4.21	3.38	3.49	9.30

Emotional clarity = Trait Meta-Mood Scale-Emotional Clarity Subscale; Self-blame = Cognitive Emotion Regulation Questionnaire-Self-blame Subscale; Other-blame = Cognitive Emotion Regulation Questionnaire-Other-blame Subscale; Rumination = Cognitive Emotion Regulation Questionnaire-Rumination Subscale; Catastrophizing = Cognitive Emotion Regulation Questionnaire-Catastrophizing Subscale; Posttraumatic stress = Posttraumatic Diagnostic Scale. *p < 0.05, **p < 0.01, ***p < 0.001.

Moreover, prior to the mediation analysis, independent sample *t*-tests and one-way analysis of variance were conducted in order to identify differences across gender and age groups in emotional clarity, maladaptive emotion regulation strategies, and posttraumatic stress symptoms. For ANOVA, Bonferroni *post hoc* testing was conducted. A significant gender difference in *self-blame* (*t* = 2.02, *p* = 0.045) and age differences in posttraumatic stress (*F* = 3.30, *p* = 0.021) were reported. Men (*M* = 8.03, SD = 3.61) were more likely to use self-blame strategies than women (*M* = 7.06, SD = 3.16). Individuals between the ages of 30 and 39 years reported the greatest posttraumatic stress symptoms (*M* = 10.72, SD = 9.91), which were significantly higher than those of individuals aged 50–69 years (*M* = 5.03, SD = 6.97). Thus, gender and age, along with factors operating before or during the earthquake, were controlled in subsequent analyses.

### Mediation Effect of Maladaptive Emotion Regulation Strategies

**FIGURE 1:**
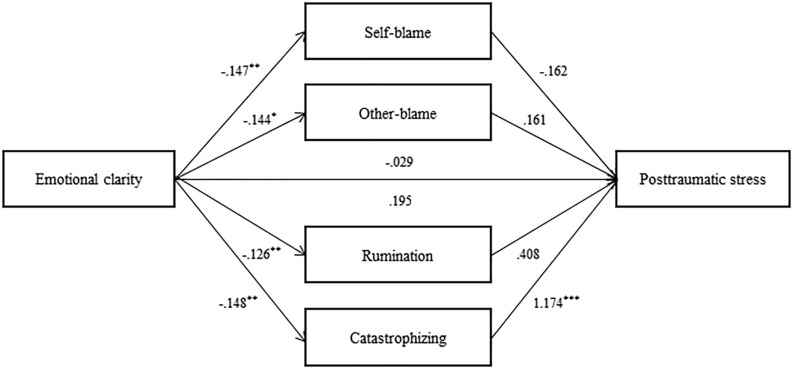
Mediation model of maladaptrve emotion regulation strategies. Unstandardized coefficient values. *p < 0.05, **p < 0.01, ***p < 0.001.

A bootstrapping procedure was conducted to examine whether maladaptive emotion regulation strategies mediate the relationship between emotional clarity and posttraumatic stress symptoms (see Figure 1). Bootstrapping was used to extract 5,000 samples, and upper and lower values of indirect effect coefficients in 95% CIs were identified. Only when 0 is not included in CI is the effect considered significant.

The total effect of emotional clarity on posttraumatic stress symptoms (*B* = −0.03, *p* = 0.812) and the direct effect after controlling for maladaptive emotion regulation strategies (*B* = 0.19, *p* = 0.064) were not significant (Table 3). The indirect effect of emotional clarity on posttraumatic stress symptoms mediated by maladaptive emotion regulation strategies was significant (CI = −0.39 to −0.08). Specifically, the mediating effect of *catastrophizing* was significant (CI = −0.29 to −0.07). However, the mediating effects of *self-blame*, *other-blame*, and *rumination* were not significant.

**TABLE 3:** Bootstrapping analysis for indirect effect of maladaptive emotion regulation strategies.

				**95% confidence**	
**Dependent variable**		***B***	**SE**	**interval**	
				**LLCI**	**ULCI**
Posttraumatic	Total indirect effect	–0.22	0.08	–0.39	–0.08
stress	Self-blame	0.02	0.04	–0.05	0.10
	Other-blame	–0.02	0.03	–0.09	0.02
	Rumination	–0.05	0.04	–0.13	0.00
	Catastrophizing	–0.17	0.06	–0.29	–0.07

*Posttraumatic stress = Posttraumatic Diagnostic Scale. LLCI: Lower level for 95% confidence interval. ULCI: Upper level for 95% confidence interval.*

## Discussion

The present study examined the mediating effect of maladaptive emotion regulation strategies on the relationship between emotional clarity and posttraumatic stress symptoms in a sample of earthquake survivors. In accordance with our hypothesis, the direct effect of emotional clarity on posttraumatic stress symptoms was not significant, while the indirect effect mediated by maladaptive emotion regulation strategies was significant, even after controlling for age, gender, and factors operating before or during the earthquake. Specifically, only catastrophizing had a significant mediating effect. This is consistent with previous findings that catastrophizing tendencies increase the risk of PTSD onset and contribute to symptom persistence following traumatic events (Bryant and Guthrie, 2005; Resick et al., 2008).

The results of the present study have implications both theoretically and clinically. First, this study examined how low emotional clarity may contribute to psychological problems such as PTSD. In situations that exceed daily stress levels, individuals who are not able to clearly identify their own emotions are apt to use maladaptive emotion regulation strategies such as catastrophizing, and this can play a role in the development of PTSD. This is consistent with the perspectives of previous studies, which suggested that the ability to identify and understand emotion is the first step in effective emotion regulation (Barrett et al., 2001; Gratz and Roemer, 2004; Mennin et al., 2007). Moreover, this supports the extended process model of emotion regulation (Gross, 2015) in which the stage of understanding or interpreting one’s emotional state is followed by the stage of selecting and implementing emotion regulation strategies.

Catastrophizing tendency assumed a significant mediating role in the present study. According to Gellatly and Beck (2016), catastrophic beliefs can contribute to PTSD by triggering interpretation bias, which increases the likelihood of negative interpretation of neutral stimuli, as well as attentional bias, which may contribute to excessive attention to threatening information. In addition, when individuals face potentially threatening stimuli, catastrophic beliefs may lead to re-experiencing of specific scenes, images, or sensations related to traumatic events. Kaczkurkin et al. (2017) also reported that catastrophizing contributes to the development and maintenance of posttraumatic stress symptoms through intrusive memories.

In clinical settings, interventions focusing on strengthening emotion identification skills may assist disaster survivors with low emotional clarity (e.g., Barlow et al., 2010). The results of this study suggest that an additional therapeutic focus should be discovering means of identifying and restructuring catastrophic thinking. Several studies have already suggested that cognitive interventions related to catastrophizing were effective for reducing PTSD symptoms because they restructure the individuals’ negative appraisal of intrusive symptoms (Ehlers and Clark, 2000; Clark and Beck, 2011).

The present study has some limitations. First, the possibility of memory bias and distortion cannot be ruled out because self-report and retrospective questionnaires were used. Because TMMS measures trait emotional clarity and CERQ assesses individuals’ typical use of cognitive emotion regulation strategies when managing unpleasant and negative experiences, they may not adequately capture survivors’ emotional clarity or emotion regulation strategies during specific moments. In addition, clinically structured interviews to assess PTSD symptoms were not conducted, and thus, it cannot be confirmed whether the reported posttraumatic stress symptoms correspond to the official diagnostic criteria for PTSD. Furthermore, external validation of emotional clarity in an experimental emotion recognition paradigm, for example, may help to overcome the limitation of self-reported emotional clarity, as well as strengthening the generalizability of the mediation effect of emotional clarity on the relationship between trauma and posttraumatic stress symptoms in the general population. Relatedly, as the ability to process emotions and/or emotional biases may also represent a trait-like quality, it will be necessary to measure and control for possible emotional biases that may affect our findings. Second, the cross-sectional design of the present study does not allow for inference regarding a causal relationship among the variables. A longitudinal study is required to explore the change in symptoms following disasters and to investigate the causal role of emotional clarity and maladaptive emotion regulation strategies. Third, participants in the current study were not grouped based on their level of trauma. This was, in part, due to the limited sample size, which was insufficient to secure a valid number of participants in high-trauma and low-trauma groups. In future studies, it will be important to replicate the current findings by further examining the mediation model based on a larger sample of participants who report a high versus low degree of trauma. Lastly, we were unable to obtain information regarding the extent of clinical services received immediately following the earthquakes and subsequent resources obtained from the community. Therefore, the effect of environmental and social factors on the participants’ degree of emotional clarity and employment of emotional regulation strategies cannot be ruled out. Future studies must consider such factors in order to more comprehensively understand the development and maintenance of PTSD in disaster survivors.

Despite such limitations, this study adds to the literature in that emotional clarity and maladaptive emotion regulation strategies were simultaneously addressed while factors operating before or during the earthquake were controlled. Most of all, our findings highlight the potential significance of emotional clarity in the chronic stage of PTSD, specifically in survivors of natural disasters such as earthquakes. Moreover, replication of the current study after addressing a number of limitations is needed, in addition to examining the effectiveness of interventions that focus on both emotional clarity and catastrophizing in a clinical sample.

## Data Availability Statement

The datasets generated for this study are available on request to the corresponding author.

## Ethics Statement

The studies involving human participants were reviewed and approved by Institutional Review Board Yonsei University. The ethics committee waived the requirement of written informed consent for participation.

## Author Contributions

HK designed the study, collected data, analyzed data, and led the drafting of the manuscript. YE participated in the rewriting of the manuscript and consulted on the research design. SP served as the principal investigator of the grant and supervised the findings of this work. All authors provided critical feedback, revised the analytic methods and manuscript, and approved the final submission.

## Conflict of Interest

The authors declare that the research was conducted in the absence of any commercial or financial relationships that could be construed as a potential conflict of interest.

## References

[B1] Ahn H. N. Lee N. B. Joo H. S. (2013). Validation of the Cognitive Emotion Regulation Questionnaire in a Korean population. *Kor. J. Couns.* 14 1773–1794. 10.15703/kjc.14.3.201306.1773

[B2] Amstadter A. B. Vernon L. L. (2008). A preliminary examination of thought suppression, emotion regulation, and coping in a trauma-exposed sample. *J. Aggress. Maltreat. Trauma* 17 279–295. 10.1080/10926770802403236 20046535 PMC2800361

[B3] Bardeen J. R. Kumpula M. J. Orcutt H. K. (2013). Emotion regulation difficulties as a prospective predictor of posttraumatic stress symptoms following a mass shooting. *J. Anxiety Disord.* 27 188–196. 10.1016/j.janxdis.2013.01.003 23454838 PMC3628280

[B4] Barlow D. H. Ellard K. K. Fairholme C. P. Farchione T. J. Boisseau C. L. May J. T. E. (2010). *Unified Protocol for Transdiagnostic Treatment of Emotional Disorders: Workbook.* Oxford: Oxford University Press.

[B5] Barrett L. F. Gross J. Christensen T. C. Benvenuto M. (2001). Knowing what you’re feeling and knowing what to do about it: mapping the relation between emotion differentiation and emotion regulation. *Cogn. Emot.* 15 713–724. 10.1080/02699930143000239

[B6] Boden M. T. Bonn-Miller M. O. Kashdan T. B. Alvarez J. Gross J. J. (2012). The interactive effects of emotional clarity and cognitive reappraisal in posttraumatic stress disorder. *J. Anxiety Disord.* 26 233–238. 10.1016/j.janxdis.2011.11.007 22169054

[B7] Boden M. T. Thompson R. J. (2015). Facets of emotional awareness and associations with emotion regulation and depression. *Emotion* 15 399–410. 10.1037/emo0000057 25706832

[B8] Bonanno G. A. Brewin C. R. Kaniasty K. Greca A. M. L. (2010). Weighing the costs of disaster: consequences, risks, and resilience in individuals, families, and communities. *Psychol. Sci. Public Interest* 11 1–49. 10.1177/1529100610387086 26168411

[B9] Brewin C. R. Andrews B. Valentine J. D. (2000). Meta-analysis of risk factors for posttraumatic stress disorder in trauma-exposed adults. *J. Consult. Clin. Psychol.* 68 748–766. 10.1037/0022-006X.68.5.748 11068961

[B10] Bryant R. A. Guthrie R. M. (2005). Maladaptive appraisals as a risk factor for posttraumatic stress: a study of trainee firefighters. *Psychol. Sci.* 16 749–752. 10.1111/j.1467-9280.2005.01608.x 16181434

[B11] Clark D. A. Beck A. T. (2011). *Cognitive Therapy of Anxiety Disorders: Science and Practice.* New York, NY: Guilford Press, NY.

[B12] Eftekhari A. Zoellner L. A. Vigil S. A. (2009). Patterns of emotion regulation and psychopathology. *Anxiety Stress Coping* 22 571–586. 10.1080/10615800802179860 19381989 PMC3234115

[B13] Ehlers A. Clark D. M. (2000). A cognitive model of posttraumatic stress disorder. *Behav. Res. Ther.* 38 319–345. 10.1016/S0005-7967(99)00123-0 10761279

[B14] Foa E. B. Cashman L. Jaycox L. Perry K. (1997). The validation of a self-report measure of posttraumatic stress disorder: the posttraumatic diagnostic scale. *Psychol. Assess.* 9 445–451. 10.1037/1040-3590.9.4.445

[B15] Garnefski N. Kraaij V. (2007). The cognitive emotion regulation questionnaire. *Eur. J. Psychol. Assess.* 23 141–149. 10.1027/1015-5759.23.3.141

[B16] Gellatly R. Beck A. T. (2016). Catastrophic thinking: a transdiagnostic process across psychiatric disorders. *Cogn. Ther. Res.* 40 441–452. 10.1007/s10608-016-9763-3

[B17] Gratz K. L. Roemer L. (2004). Multidimensional assessment of emotion regulation and dysregulation: development, factor structure, and initial validation of the difficulties in emotion regulation scale. *J. Psychopathol. Behav. Assess.* 26 41–54. 10.1023/b:joba.0000007455.08539.94

[B18] Gross J. J. (2015). Emotion regulation: current status and future prospects. *Psychol. Inq.* 26 1–26. 10.1080/1047840x.2014.940781

[B19] Hayes A. F. (2013). *Introduction to Mediation, Moderation, and Conditional Process Analysis: A Regression-Based Approach.* New York, NY: Guilford Press.

[B20] Kaczkurkin A. N. Zang Y. Gay N. G. Peterson A. L. Yarvis J. S. Borah E. V. (2017). Cognitive emotion regulation strategies associated with the DSM-5 posttraumatic stress disorder criteria. *J. Trauma. Stress* 30 343–350. 10.1002/jts.22202 28665526

[B21] Kennedy L. A. Cohen T. R. Panter A. T. Devellis B. M. Yamanis T. J. Jordan J. M. (2010). Buffering against the emotional impact of pain: mood clarity reduces depressive symptoms in older adults. *J. Soc. Clin. Psychol.* 29 975–987. 10.1521/jscp.2010.29.9.975

[B22] Lee S. J. Lee H. K. (1997). The research on the validation of the Trait Meta-Mood Scale: the domain exploration of the emotional intelligence. *Kor. J. Soc. Pers. Psychol.* 11 95–116.

[B23] Mennin D. S. (2005). “Emotion and the acceptance-based approaches to the anxiety disorders,” in *Acceptance and Mindfulness-Based Approaches to Anxiety*, eds Orsillo S. M. Roemer L. (Boston, MA: Springer), 37–68. 10.1007/0-387-25989-9_2

[B24] Mennin D. S. Holaway R. M. Fresco D. M. Moore M. T. Heimberg R. G. (2007). Delineating components of emotion and its dysregulation in anxiety and mood psychopathology. *Behav. Ther.* 38 284–302. 10.1016/j.beth.2006.09.001 17697853

[B25] Ministry of the Interior and Safety (2018). *2017 Pohang Earthquake Whitepaper.* Seoul: Ministry of the Interior and Safety.

[B26] Myers D. G. Wee D. F. (2005). *Disaster Mental Health Services: A Primer for Practitioners.* London: Psychology Press.

[B27] Nam B. R. Kwon H. I. Kwon J. H. (2010). Psychometric qualities of the Korean version of the Posttraumatic Diagnosis Scale (PDS-K). *Kor. J. Clin. Psychol.* 29 147–167. 10.15842/kjcp.2010.29.1.009

[B28] Neria Y. Nandi A. Galea S. (2008). Post-traumatic stress disorder following disasters: a systematic review. *Psychol. Med.* 38 467–480. 10.1017/s0033291707001353 17803838 PMC4877688

[B29] Ozer E. J. Best S. R. Lipsey T. L. Weiss D. S. (2003). Predictors of posttraumatic stress disorder and symptoms in adults: a meta-analysis. *Psychol. Bull.* 129 52–73. 10.1037//0033-2909.129.1.52 12555794

[B30] Resick P. A. Monson C. M. Chard K. M. (2008). *Cognitive Processing Therapy: Veteran/Military Version.* Washington, DC: U.S. Department of Veterans Affairs.

[B31] Salovey P. Mayer J. D. Goldman S. L. Turvey C. Palfai T. P. (1995). “Emotional attention, clarity and repair: exploring emotional intelligence using the Trait Meta-Mood Scale,” in *Emotion, Disclosure and Health*, ed. Pennebaker J. W. (Washington, DC: American Psychological Association), 125–154. 10.1037/10182-006

[B32] Shipman K. Edwards A. Brown A. Swisher L. Jennings E. (2005). Managing emotion in a maltreating context: a pilot study examining child neglect. *Child Abuse Negl.* 29 1015–1029. 10.1016/j.chiabu.2005.01.006 16159666

[B33] Stange J. P. Alloy L. B. Flynn M. Abramson L. Y. (2013). Negative inferential style, emotional clarity, and life stress: integrating vulnerabilities to depression in adolescence. *J. Clin. Child Adolesc. Psychol.* 42 508–518. 10.1080/15374416.2012.743104 23215673 PMC3596490

[B34] Trautmann S. Rehm J. Wittchen H. U. (2016). The economic costs of mental disorders: do our societies react appropriately to the burden of mental disorders? *EMBO Rep.* 17 1245–1249. 10.15252/embr.201642951 27491723 PMC5007565

[B35] Tull M. T. Barrett H. M. McMillan E. S. Roemer L. (2007). A preliminary investigation of the relationship between emotion regulation difficulties and posttraumatic stress symptoms. *Behav. Ther.* 38 303–313. 10.1016/j.beth.2006.10.001 17697854

[B36] Tull M. T. Hahn K. S. Evans S. D. Salters-Pedneault K. Gratz K. L. (2011). Examining the role of emotional avoidance in the relationship between posttraumatic stress disorder symptom severity and worry. *Cogn. Behav. Ther.* 40 5–14. 10.1080/16506073.2010.515187 21337211

[B37] Viana A. G. Hanna A. E. Woodward E. C. Raines E. M. Paulus D. J. Berenz E. C. (2018). Emotional clarity, anxiety sensitivity, and PTSD symptoms among trauma-exposed inpatient adolescents. *Child Psychiatry Hum. Dev.* 49 146–154. 10.1007/s10578-017-0736-x 28536961

[B38] Vine V. Aldao A. (2014). Impaired emotional clarity and psychopathology: a transdiagnostic deficit with symptom-specific pathways through emotion regulation. *J. Soc. Clin. Psychol.* 33 319–342. 10.1521/jscp.2014.33.4.319

[B39] Vine V. Marroquín B. (2018). Affect intensity moderates the association of emotional clarity with emotion regulation and depressive symptoms in unselected and treatment-seeking samples. *Cognit. Ther. Res.* 42 1–15. 10.1007/s10608-017-9870-9 29657347 PMC5894863

